# Impact of Scientific Versus Emotional Wording of Patient Questions on Doctor-Patient Communication in an Internet Forum: A Randomized Controlled Experiment with Medical Students

**DOI:** 10.2196/jmir.4597

**Published:** 2015-11-25

**Authors:** Martina Bientzle, Jan Griewatz, Joachim Kimmerle, Julia Küppers, Ulrike Cress, Maria Lammerding-Koeppel

**Affiliations:** ^1^Leibniz-Institut für Wissensmedien / Knowledge Media Research CenterKnowledge Construction LabTuebingenGermany; ^2^Competence Centre for University Teaching in MedicineUniversity of TuebingenTuebingenGermany; ^3^Department of PsychologyUniversity of TuebingenTuebingenGermany

**Keywords:** medicine, medical education, communication, Internet, counseling

## Abstract

**Background:**

Medical expert forums on the Internet play an increasing role in patient counseling. Therefore, it is important to understand how doctor-patient communication is influenced in such forums both by features of the patients or advice seekers, as expressed in their forum queries, and by characteristics of the medical experts involved.

**Objective:**

In this experimental study, we aimed to examine in what way (1) the particular wording of patient queries and (2) medical experts’ therapeutic health concepts (for example, beliefs around adhering to a distinctly scientific understanding of diagnosis and treatment and a clear focus on evidence-based medicine) impact communication behavior of the medical experts in an Internet forum.

**Methods:**

Advanced medical students (in their ninth semester of medical training) were recruited as participants. Participation in the online forum was part of a communication training embedded in a gynecology course. We first measured their biomedical therapeutic health concept (hereinafter called “biomedical concept”). Then they participated in an online forum where they answered fictitious patient queries about mammography screening that either included scientific or emotional wording in a between-group design. We analyzed participants’ replies with regard to the following dimensions: their use of scientific or emotional wording, the amount of communicated information, and their attempt to build a positive doctor-patient relationship.

**Results:**

This study was carried out with 117 medical students (73 women, 41 men, 3 did not indicate their sex). We found evidence that both the wording of patient queries and the participants’ biomedical concept influenced participants’ response behavior. They answered emotional patient queries in a more emotional way (mean 0.92, SD 1.02) than scientific patient queries (mean 0.26, SD 0.55; *t*_74_=3.48, *P*<.001, *d*=0.81). We also found a significant interaction effect between participants’ use of scientific or emotional wording and type of patient query (*F*_2,74_=10.29, *P*<.01, partial η^2^=0.12) indicating that participants used scientific wording independently of the type of patient query, whereas they used emotional wording particularly when replying to emotional patient queries. In addition, the more pronounced the medical experts’ biomedical concept was, the more scientifically (adjusted β=.20; *F*_1,75_=2.95, *P*=.045) and the less emotionally (adjusted β=–.22; *F*_1,74_=3.66, *P*=.03) they replied to patient queries. Finally, we found that participants’ biomedical concept predicted their engagement in relationship building (adjusted β=–.26): The more pronounced their biomedical concept was, the less they attempted to build a positive doctor-patient relationship (*F*_1,74_=5.39, *P*=.02).

**Conclusions:**

Communication training for medical experts could aim to address this issue of recognizing patients’ communication styles and needs in certain situations in order to teach medical experts how to take those aspects adequately into account. In addition, communication training should also make medical experts aware of their individual therapeutic health concepts and the consequential implications in communication situations.

## Introduction

Internet communication enables both medical experts [1-5] and laypeople [3-6] to perceive as well as produce and communicate health-related information online. At the same time, knowledge in the field of health and medicine is very complex, often contradictory, highly sophisticated [7,8], and therefore often difficult to evaluate. One way in which laypeople deal with this situation is to consult medical experts in expert online forums [9-13]. Although their legal assessment is disputed, medical expert forums are increasingly used for purposes of counseling patients and other people who seek medical advice [13-18]. It is characteristic of medical expert forums that patients and doctors have never seen each other before and, therefore, have no established doctor-patient relationship. This is relevant because a good doctor-patient relationship has a significant impact on various variables that support therapeutic success [19], such as patient adherence [20,21] or informed decision making [22].

Medical expert forums as a special communication channel create new challenges for medical experts arising from that particular kind of communication. Such text-based communication differs strongly from real-life counseling situations. This includes reduced audiovisual and social context clues [23-25]. Often, there is only a short period of time to establish a trustful relationship between physicians and patients; however, in computer-mediated situations, people need even more time to establish a trustful relationship than in face-to-face scenarios [26]. In addition, medical experts have to communicate with unknown and largely anonymous patients. So far, the factors have been strongly underexposed that might have an impact on how medical experts manage to be responsive to advice seekers in text-based Internet forums. Because online consultation should fulfill the same criteria of doctor-patient communication as in real-life counseling (in particular, information exchange and relationship building [27,28]), it is highly relevant to examine how potential factors of influence affect these aspects.

Previous research about medical expert forums has shown that patients consult an Internet physician for many different reasons. Typically, patients want to receive both general and individualized information as well as professional advice and/or emotional support [9,10,29,30]. So far, some studies have been published about patient expectations [9,13,29] and patient communication behavior [30-33] in health-related online forums. But very little is known about how features of the advice seekers, as expressed in their forum queries, and characteristics of the medical experts influence information exchange and relationship building between patients and doctors. Most research on expert forums has been merely based on surveys [14,15,29] or qualitative analyses of field observations [13,30,31,33]. In this study, we address this research gap both methodologically and with regard to content by investigating the communication of medical experts in an online forum in a randomized controlled experiment.

In the following sections, we discuss the potential impact of both patients’ and doctors’ characteristics on the experts’ communication behavior. In particular, we consider the role of the wording of patient queries in terms of scientific versus emotional phrasing and of the experts’ beliefs about medicine (ie, their individual therapeutic health concept).

### Impact of Patient Queries

As in all text-based communication, an expert online forum offers a reduced communication setting [25] in which the post or query of another user is the crucial external stimulus that triggers the communication process. For this reason, the wording of the queries may play a very important role. It is plausible that the response behavior of medical experts in such forums is affected by the patient requests themselves. In communicating with patients, the capability of a medical expert to adapt his or her own communication style to that of a patient is an important conversational skill. Research about tailored health communication shows that the fit between health messages and the individual characteristics of patients or advice seekers influences how they handle those messages [34-39]. Tailored messages are processed more deeply, for instance [36], and are better learned [34] than nontailored messages. This illustrates the great theoretical and practical relevance of this aspect of communication.

It is well established that people often imitate the behavior [40] and the communication style of their interaction partners [41,42]. This is particularly conveyed in people’s ability to mimic emotional expressions [43-45]. Recent research has found that laypeople tend to reply more emotionally to forum queries containing personal experiences than to fact-oriented queries [32]. In conversation research, the concept of *lexical entrainment* [46-48] refers to overlaps between conversational partners in their choice of words. Research about written communication between medical experts and laypeople found that the answer of a medical expert to a patient query was influenced by the word choice in the query [46,48]. Accordingly, medical experts are also assumed to apply a conversation technique of imitating conversational partners’ word choice to text-based communication in expert online forums. Thus, we expect that medical experts will reply in a more emotional way to emotional patient queries (Hypothesis 1a) and in a more scientific way to scientific patient queries (Hypothesis 1b).

### Impact of Medical Experts’ Therapeutic Health Concept

In addition to the impact of the communication partner, the communication process is also influenced by individual characteristics, such as personal values, attitudes, or beliefs. Medical experts’ beliefs about medicine (ie, about the relevant aspects of health, diagnosis, and therapy in medicine) are strongly triggered by the *International Classification of Diseases* (*ICD*), which is the most common classification catalog in conventional medicine [49]. The *ICD* is clearly based on a biomedical conception of diagnostic and therapeutic approaches. A biomedically oriented therapeutic health concept (hereinafter called “biomedical concept”) adheres to a distinctly scientific understanding of diagnosis and treatment and a clear focus on evidence-based medicine [50-52]. At the same time, it is occasionally recorded that personal and emotional aspects of doctor-patient relationships are largely disregarded in communication that is derived from the biomedical perspective [53]. Medical students, for instance, state that they feel insufficiently prepared regarding psychosocial skills [54]. Even though psychology is considered to be an integral part of medical professionalization, psychosocial aspects are often not regarded as important from a biomedical point of view [55]. We assume that the strength of the individual biomedical concept of medical experts will have an impact on their communication style in patient counseling. This should apply to communication in online forums as well. Accordingly, we expect that the more pronounced the medical experts’ biomedical concept, the more scientifically they will reply to patient queries (Hypothesis 2a). We also assume that the more pronounced the medical experts’ biomedical concept, the less emotionally they will reply to patient queries (Hypothesis 2b).

### Interaction Effects of Patient Queries and Medical Experts’ Biomedical Health Concept

An interesting research question is whether the style of patient requests and the medical experts’ biomedical concept will interact in having an impact on physician-patient communication. Individuals tend to be more attracted to people who they perceive to have similar attitudes or beliefs to their own [56,57]; for example, when patients perceive similarities between themselves and their doctor, they are more satisfied with the medical care [58]. There is also a positive relationship between information-sharing behavior and similarity [59]. It has been found that information transfer is more likely between individuals who display similar attitudes. Accordingly, we assume that medical experts with a pronounced biomedical concept who encounter patient queries that express some sort of like-mindedness (ie, include scientific wording) will be willing to provide more information to those patients than to others without that fit (Hypothesis 3).

Another important aspect of a doctor-patient interaction is the development of a positive relationship between physicians and patients [27]. With regard to the restrictions of text-based communication in an online forum (eliminating large parts of nonverbal and paraverbal communication channels), one means of relationship building for physicians is to verbally express their respect and acceptance of a communication partner, for instance, through courtesy or polite expressions of salutation and valediction [60,61]. In line with our previous reasoning, we assume that perceived similarity will also have an impact on that kind of communication behavior. Therefore, we expect that medical experts with a pronounced biomedical concept who encounter scientifically phrased patient queries will engage more strongly in relationship building than without that fit (Hypothesis 4).

### This Study

We set up an experimental study to investigate how advanced medical students with a more- or less-pronounced biomedical concept reacted to patient queries presented to them in an expert online forum. Those patient queries had either scientific or emotional wording. The goal of the study was to examine how the particular wording of the patient queries and the strength of the medical students’ biomedical concepts affected participants’ response behavior in the online forum. We also aimed to examine how the biomedical concept and the patient queries would interact. Outcome variables of interest were (1) participants’ use of scientific and emotional wording, (2) the amount of communicated information, and (3) engagement in relationship building in their reply posts.

We created an online forum that participants used as a part of communication training that was embedded in a gynecology class. This forum was developed using the open-source server-side scripting language PHP. It was divided into a shared discussion forum and individual (password-protected) subforums where participants had the opportunity to respond to patient queries. For the study, each participant replied to a particular patient query about mammography screening in her or his individual subforum (time limit for reply: 15 minutes). These queries either included scientific or emotional wording in a between-group design.

## Methods

### Sample

This study was carried out with 117 medical students in the Faculty of Medicine of the University of Tuebingen (Germany). All participants were students in the ninth semester of medical training. All medical students in the ninth semester who attended the communication training were allowed to participate in the study because this was part of their gynecology education. We considered advanced medical students to be medical experts [48,62] according to the definition of an expert as “a person with training in a particular field who is able to tackle complex problems because of this training and additional practical experience” (p 317 [62]). The students were divided into groups of 8 to 10 participants. Each week, one group passed through the study. The inquiry period lasted 13 weeks. In each group, half of the students were randomly assigned to one experimental condition and the other half to the other experimental condition (scientific vs emotional wording of patient queries). The randomization was ensured by a computer-generated assignment of individual log-in information (anonymous code and password). By logging in, the students were automatically guided to one of the 2 experimental conditions. At this point, participants were not aware of the fact that there were different kinds of patient queries. After the study was completed, students were debriefed by the instructor of their class.

### Procedure and Experimental Material

First, we collected demographic data. Then, we measured the strength of the participants’ biomedical concept. Subsequently, each participant responded to one fictitious patient request in the online expert forum in the doctor’s role. We recorded their individual reply posts and analyzed them regarding the use of scientific and emotional wording, the amount of communicated information, and engagement in relationship building.

Half of the participants answered patient queries that were written using scientific wording and the other half replied to a request written in an emotional style. The scientific queries included terms such as “scientifically proven,” “evidence,” and “study.” The emotional queries contained wordings such as “I am concerned,” “makes me anxious,” and “my feelings.” In order to ensure that it was not an unintended aspect of a particular query that triggered participants’ replies, we created 6 different patient queries (3 with scientific and 3 with emotional wording). The queries were comparably long (scientific queries: mean 74.67 words, SD 28.75; emotional queries: mean 81.00 words, SD 15.13). The scientific queries contained a mean 3.67 scientific words (SD 1.53) and the emotional queries contained a mean 3.00 emotional words (SD 1.00). The scientific queries did not contain any emotional words and the emotional queries did not contain any scientific words. Both types of queries asked the medical experts for information about mammography screening. With regard to content, the patient queries addressed questions such as whether and why a mammogram was suitable for their individual situation or asked about the risks and benefits of mammography and other diagnostic methods.

### Measures

To measure the strength of participants’ biomedical concept, we used the biomedical subscale of the Therapeutic Health Concepts Scale [63]. Participants were asked to rate the importance of 5 characteristic biomedical terms (diagnosis, science, evidence-based methods, standardized tests, and medical guidelines) on 6-point Likert scales ranging from 1 (not important) to 6 (very important). Internal consistency for that scale was acceptable (α=.66).

To capture the outcome variables, we analyzed the answers of the participants in coding-and-counting procedures. The coders of participants’ answers were blinded to the experimental condition. This was accomplished by downloading only the answers of the participants from the online forum—and not the corresponding patient query. This ensured that the raters had no hint as to the experimental condition. Based on a post hoc qualitative content analysis, we identified 19 scientific and 18 emotional keywords on the basis of a subset of replies. The identification of keywords was performed by the first author and validated by the third author following existing literature [49,64]. Examples of scientific keywords were “statistic,” “evident,” or “to prove.” Examples of emotional keywords were “anxiety,” “sorrow,” or “to calm.” The whole set of scientific and emotional words is presented in Multimedia Appendix 1. In our analysis, we counted how often these words were used in total in each of the answers of the individual medical students.

Regularly, several units of information were provided in one reply post, for example, information about organizational issues, about risk factors, or advice for further diagnostic course of action. In a first step, every unit of information was coded using the codes “advice,” “organizational information,” “information about mammography,” “information about other methods,” “information about breast cancer,” and “information about risk factors.” To measure the amount of information provided by the participants in their reply posts, we calculated a total score of all given units of information. Half of the reply posts were coded by 2 independent raters for units of information. Interrater reliability was *r*=.85. Because of this high level of agreement, the remaining reply posts were coded by one rater.

To measure the participants’ attempts to build a positive doctor-patient relationship, we also applied a coding-and-counting procedure. Two independent coders identified wording in which participants attended to patients’ needs and wants (eg, “If you have further questions, please do not hesitate to contact me”) and situations in which participants thanked the patients for their interest, request, or trust. Interrater reliability was *r*=.67.

### Statistical Analysis

We used 2 independent sample *t* tests to examine the impact of patient queries on the use of scientific and emotional wording, respectively. We also conducted a mixed model analysis of variance (ANOVA) with patient queries as the between-subject factor and the use of scientific and emotional wording as the within-subject factor. To examine the influence of medical experts’ biomedical concept on the use of emotional and scientific words, we calculated linear regression models. Finally, we calculated moderated regression analyses to test main and interaction effects of patient queries and biomedical concept on the amount of communicated information and on the participants’ attempt to build a positive doctor-patient relationship. The sample size was calculated to detect moderate to large effects (*d*=0.65, *f*=0.35, *f* ^*2*^=0.15) with 90% power at the 5% significance level using G*Power 3.1.7 [65]. This procedure resulted in a total sample size requirement of 84 for independent sample *t* tests, 68 for ANOVAs, and 59 for linear regression analyses.

### Ethical Considerations

This research was performed in accordance with the Declaration of Helsinki. The study was approved by the head of the gynecology program at the University Hospital Tuebingen and the Faculty of Medicine as well as by the Competence Centre for University Teaching in Medicine. All students participated voluntarily and anonymously. They gave informed consent and were informed about privacy protection, their right to terminate participation at any time without disadvantages, and about the general purpose of the study.

## Results

### Participant Characteristics

**Figure 1:**
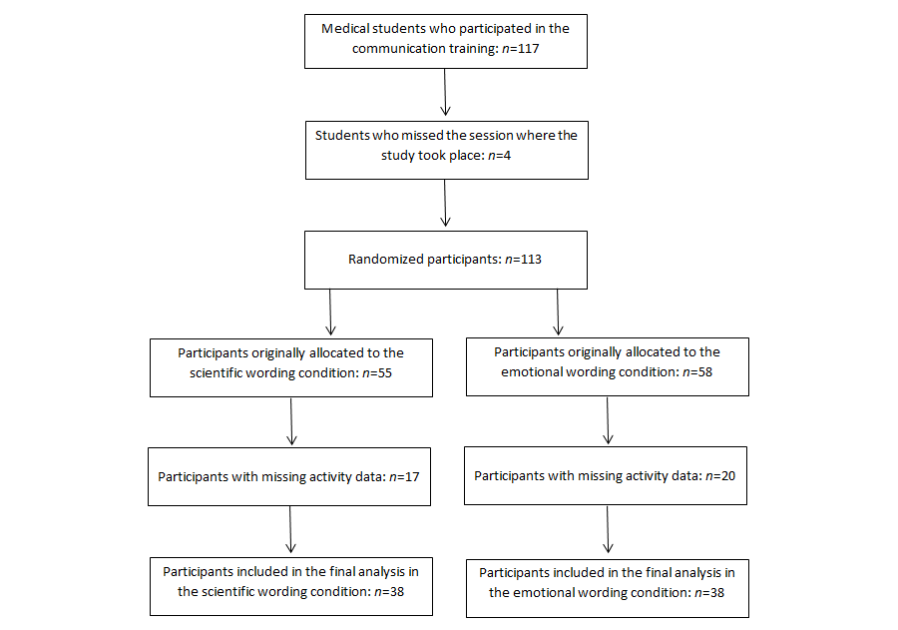
Participants’ progress through the phases of the study.

Four students missed the training session where the study took place. In addition, because of technical difficulties (eg, server failure), activity data of 37 participants were not properly recorded and could not be used for further analysis. Nevertheless, we were able to collect the demographic data of 38 of these 41 participants. Included and excluded participants did not differ regarding age (*P*=.46) or sex (*P*=.86) and both conditions were equally affected by the loss of participants. Thus, the following results refer to the data of the remaining 76 participants (potential consequences of this reduced sample are discussed subsequently). In all, 38 of those participants were assigned to the scientific and the other 38 participants were assigned to the emotional wording condition. Table 1 shows participants’ characteristics (sex and age) in both conditions as well as the characteristics of the participants with missing data. The loss of participants is also illustrated in Figure 1.

**Table 1:** Participant characteristics (N=117).

Participants		Participants in the scientific wording condition n=38	Participants in the emotional wording condition n=38	Participants with incomplete data n=41
**Sex, n**				
	Women	25	23	25
	Men	13	15	13
	N/A	—	—	3
**Age, n**				
	19-22	2	1	4
	23-26	26	24	18
	27-30	7	9	13
	>31	3	4	3
	N/A	—	—	3

### Impact of Patient Queries

In Hypothesis 1a, we assumed that medical experts would reply in a more emotional way to emotional patient queries than to scientific queries. The results supported this hypothesis (*t*_74_=3.48, *P*<.001, *d*=0.81). Emotional patient queries were answered in a more emotional way (mean 0.92, SD 1.02) than scientific patient queries (mean 0.26, SD 0.55).

According to Hypothesis 1b, we assumed that health care professionals would reply in a more scientific way to scientific patient queries than to emotional patient queries. Even though the descriptive data hinted toward this hypothesis (scientific patient queries: mean 1.11, SD 1.25; emotional patient queries: mean 0.82, SD 1.04), it was not statistically supported by the data (*t*_74_=1.10, *P*=.14).

**Figure 2:**
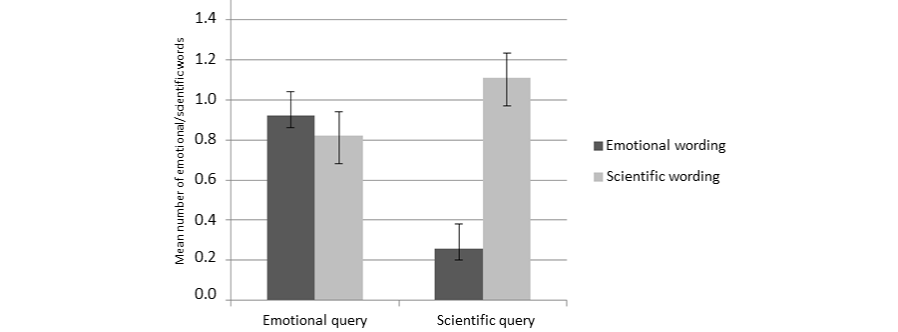
Interaction effect between type of patient query and use of scientific and emotional wording. Standard errors are represented by error bars attached to each column.

To further explore the basis of these (absent) effects, we calculated a mixed model ANOVA. We found that, in general, scientific terms were used more frequently (mean 0.96, SD 1.15) than emotional terms (mean 0.59, SD 0.88; *F*_1,74_=6.23, *P*=.02, partial η^2^=0.08). In addition, we found a significant interaction effect between use of scientific and emotional wording and the type of the patient query (*F*_2,74_=10.29, *P*=.002, partial η^2^=0.12), indicating that participants used scientific wording quite independently of the type of patient query, whereas they used emotional wording particularly in replying to emotional patient queries (see Figure 2).

### Impact of the Biomedical Concept

As a first step, we analyzed how pronounced the participants’ biomedical concept was. Their rating (mean 4.88, SD 0.63) was significantly higher than the midpoint (3.5) of the 6-point scale (*t*_75_=19.07, *P*<.001, *d*=2.19). This indicates that the participants had a strong biomedical orientation.

Hypothesis 2a stated the expectation that the more pronounced the medical experts’ biomedical concept, the more scientifically they would reply to patient queries. This hypothesis was supported by the data: a greater level of medical experts’ biomedical concept was significantly associated with using scientific terms (adjusted β=.20). A linear model with biomedical concept as predictor explained 4% of the variance (*R* ^*2*^=.04, *F*_1,75_=2.95, *P*=.045).

In Hypothesis 2b, we assumed that the more pronounced the medical experts’ biomedical concept, the less emotionally they would reply to patient queries. This hypothesis was also supported by the data: a greater level of medical experts’ biomedical concept was significantly associated with using emotional terms to a lesser extent (adjusted β=–.22). A linear model with biomedical concept as predictor explained 5% of the variance (*R* ^*2*^=.05, *F*_1,74_=3.66, *P*=.03).

### Interaction Effects of Patient Queries and Biomedical Concept

According to Hypothesis 3, we assumed that medical experts with a pronounced biomedical concept who encountered patient queries worded scientifically would be willing to provide more information to those patients than to others without that fit. To test this assumption, we conducted a moderated regression analysis. The overall model with biomedical concept, type of patient query, and the interaction term between biomedical concept and type of patient query was not significant (*R* ^*2*^=.06, *F*_3,71_=1.52, *P*=.22) and so the hypothesis did not hold.

In Hypothesis 4, we expected that medical experts with a pronounced biomedical concept who encountered scientifically phrased patient queries would engage more strongly in relationship building than they would without that fit. To test this assumption, we conducted a further regression analysis. The overall model with biomedical concept, type of patient query, and the interaction term was not significant (*F*_3,71_=2.44, *P*=.07). The overall model explained 9% of the variance (*R* ^*2*^=.09). As shown in Table 2, the biomedical concept of medical experts was the only significant predictor for relationship building: a greater level of medical experts’ biomedical concept was significantly associated with less engagement in relationship building (*r*=–.26, *P*=.01).

**Table 2:** Model showing impact of biomedical concept, type of patient query, and the interaction term between biomedical concept and type of patient query on relationship building.

Predictor	Regression coefficient	Standard error	Standardized β	*P*
Biomedical concept	–.36	0.13	–.28	.04
Patient query	.18	0.18	.11	.33
Biomedical concept × patient query	.18	0.18	.17	.34

## Discussion

### Main Findings

In this experimental study, we examined in what way the particular wording of patient queries and medical experts’ therapeutic health concepts had an impact on the communication behavior of the medical experts in an Internet forum. We found evidence that both the wording of patient queries and the strength of medical experts’ biomedical concept influenced their response behavior. Our study showed that medical experts replied in a more emotional way to emotional patient queries than to scientific patient queries. However, there was no significant main effect of scientific patient queries on scientific wording in the experts’ replies. The activity data of 41 participants were not available for the analysis; thus, the required sample size for an independent *t* test was not fully achieved. Therefore, we cannot be sure whether there was indeed no effect of scientific patient queries or whether we were not able to detect this effect due to a slightly underpowered analysis.

An interaction effect between the use of scientific and emotional wording and the type of the patient query also pointed out why there was a significant result for emotional but not for scientific queries: the participants, in their responses as medical experts, used scientific terms independently of the type of the patient query, whereas emotional terms were particularly used in replying to emotional patient queries, but seldom in replying to scientific queries. This finding shows that medical experts did not automatically adapt their communication style to that of their patients in the online forum. It seems that the participants were firmly attached to the scientific orientation and the communication style of a mainly biomedically oriented field. Seen from the patients’ perspective, it seems that patients who made their inquiry in a scientific way were more likely to receive an answer apparently adjusted to their individual point of view, whereas patients who asked in an emotional way had a better chance of receiving an answer that integrated scientific and emotional aspects. Research about the adaptation of experts’ communication style to laypeople has found that this adaptation may occur in an active, conscious, and targeted way—as it is usually in research about tailored communication [34-37]. However, this adaptation may also happen in a less conscious and rather automatic fashion. One explanation for a less conscious adaptation is the availability hypothesis, which states that the wording that is used by the conversational partner is simply easier to access and therefore used more frequently [46]. The results of our study are partly in line with the availability hypothesis, but also show that there was no complete adaptation to the communication style of the patients. If this had been the case, medical experts would have been using less scientific words when replying to emotional patient queries.

We also investigated to what extent the communication process was influenced by medical experts’ biomedical concept. We found that a stronger biomedical orientation was significantly associated with using scientific words more. Additionally, we found that a greater level of medical experts’ biomedical concept was significantly associated with using less emotional words and engaging less in relationship building. It is necessary to point out, however, that medical experts’ biomedical concept made only a small contribution to the explanation of variance. But we may conclude that even in an anonymous forum setting, medical advisors’ personal therapeutic health concepts tend to influence the communication process. This is also remarkable in view of the fact that scientific convictions are, of course, far from excluding emotional, empathetic, and relationship-oriented communication styles. But it can occasionally be observed that personal and emotional aspects of a doctor-patient relationship are largely disregarded in the biomedical perspective [54-56]. This state of affairs appears to be reflected in these results.

### Limitations and Future Work

A potential limitation of this study is that the generalizability of the findings to the whole population of medical experts and to real online forums has to be handled with care. The study was carried out with medical students and was embedded in a seminar at the Faculty of Medicine. In addition, we cannot entirely rule out that the loss of the activity data of 41 participants has potentially changed the results of our study. It has been found in previous research that a loss of participants can change the interpretation of study results [66]. It also remains unclear to what extent the results are influenced by the scientific orientation of the medical field in general and whether medical doctors with many years of working experience would show comparable response behavior to patient queries. Additionally, we cannot be sure whether medical students would answer in the same way if they were faced with patient queries in a real forum on the Internet because the behavior of individuals may be strongly influenced by their particular environment. To examine this, the response behavior of medical experts in actual online forums should be analyzed in future studies according to the criteria presented here. Another limitation is that we focused only on the biomedical concept and on one medical topic (mammography). It is possible that other health professionals, such as physiotherapists, who possess a biopsychosocial therapeutic health concept [63,67], would show different response behaviors in replying to patient queries.

Moreover, this study focused only on the response behavior of medical experts and did not consider the reception and information procession of laypeople. It would be very interesting to apply an extended study design in which laypeople with various levels of scientific orientation or with particular emotional needs would rate the experts’ answers regarding understandability, usefulness, empathy, or importance to their own decision [68,69]. In addition, one could test laypeople’s knowledge acquisition after reading the reply posts in order to examine whether they learn more from particular wordings or understand them more easily. It would also be relevant for future studies to consider other particular features of the quality of the information provided by medical experts in online forums, for instance, correct versus incorrect information. Finally, it would be interesting to examine how biomedically oriented medical experts would respond to scientific or emotional queries in alternative medical online forums [70,71].

### Conclusions

This study points out that the particular wording of patient queries and medical experts’ therapeutic health concept had an impact on the communication behavior of the medical experts in an Internet forum. The results of the study demonstrated that advanced medical students already possessed key competences in Web-based communication with laypeople. We found that the participants replied in a more emotional way to emotional patient queries than to scientific patient queries. The result that they always used scientific terms—independently of the type of patient query—could be understood as an indispensable course of action in communicating about an evidence-based procedure such as mammography screening. This finding, however, could also be interpreted as a kind of communication deficit because medical experts did not entirely adapt their communication style to that of their patients in the online forum. In addition, we found that even in an anonymous forum setting, medical advisors’ personal therapeutic health concepts tend to influence the communication process. A greater orientation toward a biomedical perspective was significantly associated with using scientific words to a higher degree and using emotional terms to a lesser degree. This shows that medical experts tended to adhere to their own science-oriented communication style in conversations with laypeople. Finally, we found that medical experts who were highly oriented toward biomedicine engaged in relationship building to a lower degree than medical experts who had less orientation toward biomedicine. Accordingly, communication training for medical experts could set addressing the issue of recognizing patients’ communication style and needs in a given situation as a learning objective in order to teach them how to take those aspects into account adequately. Furthermore, communication training should include making medical experts aware of their individual therapeutic health concept and the implications of having that concept in communication situations. On the whole, communication training should emphasize the importance of providing scientific, evidence-based information adapted to patients’ particular communication styles in a perceptive manner, regardless of the medical expert’s own therapeutic health concept.

## Multimedia Appendix 1

List of scientific and emotional wordings in participants’ reply posts.

## Abbreviations

ICD: International Classification of Diseases

## References

[1] Streeter JL, Lu MT, Rybicki FJ (2007). Informatics in radiology: RadiologyWiki.org: the free radiology resource that anyone can edit. Radiographics.

[2] von Muhlen M, Ohno-Machado L (2012). Reviewing social media use by clinicians. J Am Med Inform Assoc.

[3] van der Eijk M, Faber MJ, Aarts JW, Kremer JA, Munneke M, Bloem BR (2013). Using online health communities to deliver patient-centered care to people with chronic conditions. J Med Internet Res.

[4] Archambault PM, Turgeon AF, Witteman HO, Lauzier F, Moore L, Lamontagne F, Horsley T, Gagnon M, Droit A, Weiss M, Tremblay S, Lachaine J, Le Sage N, Émond M, Berthelot S, Plaisance A, Lapointe J, Razek T, van de Belt TH, Brand K, Bérubé M, Clément J, Grajales III FJ, Eysenbach G, Kuziemsky C, Friedman D, Lang E, Muscedere J, Rizoli S, Roberts DJ, Scales DC, Sinuff T, Stelfox HT, Gagnon I, Chabot C, Grenier R, Légaré F, Canadian Critical Care Trials Group (2015). Implementation and evaluation of a wiki involving multiple stakeholders including patients in the promotion of best practices in trauma care: the WikiTrauma Interrupted Time Series Protocol. JMIR Res Protoc.

[5] Archambault PM, van de Belt TH, Grajales FJ, Faber MJ, Kuziemsky CE, Gagnon S, Bilodeau A, Rioux S, Nelen WL, Gagnon M, Turgeon AF, Aubin K, Gold I, Poitras J, Eysenbach G, Kremer JA, Légaré F (2013). Wikis and collaborative writing applications in health care: a scoping review. J Med Internet Res.

[6] Eysenbach G, Powell J, Englesakis M, Rizo C, Stern A (2004). Health related virtual communities and electronic support groups: systematic review of the effects of online peer to peer interactions. BMJ.

[7] Sniderman AD, Furberg CD (2009). Why guideline-making requires reform. JAMA.

[8] Kienhues D, Stadtler M, Bromme R (2011). Dealing with conflicting or consistent medical information on the web: When expert information breeds laypersons' doubts about experts. Learning and Instruction.

[9] Briet JP, Hageman MG, Blok R, Ring D (2014). When do patients with hand illness seek online health consultations and what do they ask?. Clin Orthop Relat Res.

[10] Umefjord G, Petersson G, Hamberg K (2003). Reasons for consulting a doctor on the Internet: Web survey of users of an Ask the Doctor service. J Med Internet Res.

[11] Rhebergen MD, Lenderink AF, van Dijk FJ, Hulshof CT (2012). Comparing the use of an online expert health network against common information sources to answer health questions. J Med Internet Res.

[12] Richter JG, Becker A, Schalis H, Koch T, Willers R, Specker C, Monser R, Schneider M (2011). An ask-the-expert service on a rheumatology web site: who were the users and what did they look for?. Arthritis Care Res (Hoboken).

[13] Umefjord G, Sandström H, Malker H, Petersson G (2008). Medical text-based consultations on the Internet: a 4-year study. Int J Med Inform.

[14] Nijland N, van Gemert-Pijnen JE, Boer H, Steehouder MF, Seydel ER (2009). Increasing the use of e-consultation in primary care: results of an online survey among non-users of e-consultation. Int J Med Inform.

[15] Beckjord EB, Finney Rutten LJ, Squiers L, Arora NK, Volckmann L, Moser RP, Hesse BW (2007). Use of the internet to communicate with health care providers in the United States: estimates from the 2003 and 2005 Health Information National Trends Surveys (HINTS). J Med Internet Res.

[16] Chou WS, Hunt YM, Beckjord EB, Moser RP, Hesse BW (2009). Social media use in the United States: implications for health communication. J Med Internet Res.

[17] Kontos E, Blake KD, Chou WS, Prestin A (2014). Predictors of eHealth usage: insights on the digital divide from the Health Information National Trends Survey 2012. J Med Internet Res.

[18] Crotty BH, Tamrat Y, Mostaghimi A, Safran C, Landon BE (2014). Patient-to-physician messaging: volume nearly tripled as more patients joined system, but per capita rate plateaued. Health Aff (Millwood).

[19] Stewart MA (1995). Effective physician-patient communication and health outcomes: a review. CMAJ.

[20] Zolnierek KB, Dimatteo MR (2009). Physician communication and patient adherence to treatment: a meta-analysis. Med Care.

[21] Swain S, Hariharan M, Rana S, Chivukula U, Thomas M (2014). Doctor-patient communication: impact on adherence and prognosis among patients with primary hypertension. Psychol Stud.

[22] Ha JF, Longnecker N (2010). Doctor-patient communication: a review. Ochsner J.

[23] Dubrovsky V, Kiesler S, Sethna B (1991). The equalization phenomenon: status effects in computer-mediated and face-to-face decision-making groups. Human-Comp. Interaction.

[24] Sproull L, Kiesler S (1986). Reducing social context cues: electronic mail in organizational communication. Management Science.

[25] Kiesler S, Siegel J, McGuire TW (1984). Social psychological aspects of computer-mediated communication. American Psychologist.

[26] Wilson JM, Straus SG, McEvily B (2006). All in due time: the development of trust in computer-mediated and face-to-face teams. Organizational Behavior and Human Decision Processes.

[27] Ong LM, de Haes JC, Hoos AM, Lammes FB (1995). Doctor-patient communication: a review of the literature. Soc Sci Med.

[28] Parker J, Thorson E (2009). Health Communication in the New Media Landscape.

[29] Himmel W, Meyer J, Kochen MM, Michelmann HW (2005). Information needs and visitors' experience of an Internet expert forum on infertility. J Med Internet Res.

[30] Vennik FD, Adams SA, Faber MJ, Putters K (2014). Expert and experiential knowledge in the same place: patients' experiences with online communities connecting patients and health professionals. Patient Educ Couns.

[31] Attard A, Coulson NS (2012). A thematic analysis of patient communication in Parkinson’s disease online support group discussion forums. Computers in Human Behavior.

[32] Kimmerle J, Bientzle M, Cress U (2014). Personal experiences and emotionality in health-related knowledge exchange in Internet forums: a randomized controlled field experiment comparing responses to facts vs personal experiences. J Med Internet Res.

[33] Coulson NS, Buchanan H, Aubeeluck A (2007). Social support in cyberspace: a content analysis of communication within a Huntington's disease online support group. Patient Educ Couns.

[34] Albada A, Ausems MG, Bensing JM, van Dulmen S (2009). Tailored information about cancer risk and screening: a systematic review. Patient Educ Couns.

[35] Hawkins RP, Kreuter M, Resnicow K, Fishbein M, Dijkstra A (2008). Understanding tailoring in communicating about health. Health Educ Res.

[36] Kreuter MW, Wray RJ (2003). Tailored and targeted health communication: strategies for enhancing information relevance. Am J Health Behav.

[37] Rimer BK, Kreuter MW (2006). Advancing tailored health communication: a persuasion and message effects perspective. J Communication.

[38] Dowsett SM, Saul JL, Butow PN, Dunn SM, Boyer MJ, Findlow R, Dunsmore J (2000). Communication styles in the cancer consultation: preferences for a patient-centred approach. Psychooncology.

[39] Rodin G, Mackay JA, Zimmermann C, Mayer C, Howell D, Katz M, Sussman J, Brouwers M (2009). Clinician-patient communication: a systematic review. Support Care Cancer.

[40] Chartrand TL, Lakin JL (2013). The antecedents and consequences of human behavioral mimicry. Annu Rev Psychol.

[41] Moore R (2012). Imitation and conventional communication. Biol Philos.

[42] Seitz S, Stewart C (1975). Imitation and expansions: some developmental aspects of mother-child communications. Developmental Psychology.

[43] Häfner M, Ijzerman H (2011). The face of love: spontaneous accommodation as social emotion regulation. Pers Soc Psychol Bull.

[44] van der Velde SW, Stapel DA, Gordijn EH (2009). Imitation of emotion: When meaning leads to aversion. Eur J Soc Psychol.

[45] Wallbott HG (1991). Recognition of emotion from facial expression via imitation? Some indirect evidence for an old theory. Br J Soc Psychol.

[46] Jucks R, Becker B, Bromme R (2008). Lexical entrainment in written discourse: is experts' word use adapted to the addressee?. Discourse Processes.

[47] Brennan SE, Clark HH (1996). Conceptual pacts and lexical choice in conversation. J Exp Psychol Learn Mem Cogn.

[48] Bromme R, Jucks R, Wagner T (2005). How to refer to ‘diabetes’? Language in online health advice. Appl Cognit Psychol.

[49] World Health Organization (1992). International Statistical Classification of Disease and Related Health Problems, tenth revision.

[50] Engel GL (1960). A unified concept of health and disease. Perspect Biol Med.

[51] Engel GL (1977). The need for a new medical model: a challenge for biomedicine. Science.

[52] Bensing J (2000). Bridging the gap. The separate worlds of evidence-based medicine and patient-centered medicine. Patient Educ Couns.

[53] Roter DL, Stewart M, Putnam SM, Lipkin M, Stiles W, Inui TS (1997). Communication patterns of primary care physicians. JAMA.

[54] Jungbauer J, Alfermann D, Kamenik C, Brähler E (2003). [Psychosocial skills training unsatisfactory results from interviews with medical school graduates from seven German universities]. Psychother Psychosom Med Psychol.

[55] Gallagher S, Wallace S, Nathan Y, McGrath D (2015). 'Soft and fluffy': medical students' attitudes towards psychology in medical education. J Health Psychol.

[56] Byrne D, Berkowitz L (1969). Attitudes and attraction. Advances in Experimental Social Psychology.

[57] Byrne D, Gouaux C, Griffitt W, Lamberth J, Murakawa N, Prasad M, Ramirez M (1971). The ubiquitous relationship: attitude similarity and attraction: a cross-cultural study. Human Relations.

[58] Street RL, O'Malley KJ, Cooper LA, Haidet P (2008). Understanding concordance in patient-physician relationships: personal and ethnic dimensions of shared identity. Ann Fam Med.

[59] Tajfel H, Billig MG, Bundy RP, Flament C (1971). Social categorization and intergroup behaviour. Eur J Soc Psychol.

[60] Williams S, Weinman J, Dale J (1998). Doctor-patient communication and patient satisfaction: a review. Fam Pract.

[61] Comstock LM, Hooper EM, Goodwin JM, Goodwin JS (1982). Physician behaviors that correlate with patient satisfaction. J Med Educ.

[62] Bromme R, Rambow R, Nückles M (2001). Expertise and estimating what other people know: the influence of professional experience and type of knowledge. J Exp Psychol Appl.

[63] Bientzle M, Cress U, Kimmerle J (2014). Epistemological beliefs and therapeutic health concepts of physiotherapy students and professionals. BMC Med Educ.

[64] Oatley K, Keltner D, Jenkins J (2006). Understanding Emotions.

[65] Faul F, Erdfelder E, Lang A, Buchner A (2007). G*Power 3: a flexible statistical power analysis program for the social, behavioral, and biomedical sciences. Behav Res Methods.

[66] Akl EA, Briel M, You JJ, Sun X, Johnston BC, Busse JW, Mulla S, Lamontagne F, Bassler D, Vera C, Alshurafa M, Katsios CM, Zhou Q, Cukierman-Yaffe T, Gangji A, Mills EJ, Walter SD, Cook DJ, Schunemann HJ, Altman DG, Guyatt GH (2012). Potential impact on estimated treatment effects of information lost to follow-up in randomised controlled trials (LOST-IT): systematic review. BMJ.

[67] Bientzle M, Cress U, Kimmerle J (2013). How students deal with inconsistencies in health knowledge. Med Educ.

[68] Bientzle M, Cress U, Kimmerle J (2015). The role of tentative decisions and health concepts in assessing information about mammography screening. Psychol Health Med.

[69] Fissler T, Bientzle M, Cress U, Kimmerle J (2015). The impact of advice seekers’ need salience and doctors’ communication style on attitude and decision making: a web-based mammography consultation role play. JMIR Cancer.

[70] Kimmerle J, Thiel A, Gerbing K, Bientzle M, Halatchliyski I, Cress U (2013). Knowledge construction in an outsider community: extending the communities of practice concept. Computers in Human Behavior.

[71] Kimmerle J, Gerbing KK, Cress U, Thiel A (2012). Exchange of complementary and alternative medical knowledge in sport-related Internet fora. Sociol Sport J.

