# Supplementary material for: Impact of Scientific Versus Emotional Wording of Patient Questions on Doctor-Patient Communication in an Internet Forum: A Randomized Controlled Experiment with Medical Students
Source: J Med Internet Res. 2015 Nov 25;17(11):e268. doi: 10.2196/jmir.4597 (PMC4704952; doi:10.2196/jmir.4597)
Supplement: Supplementary file 1 [file jmir_v17i11e268_app1.pdf]

Multimedia Appendix 1. List of scientific and emotional wordings in participants' reply posts.

**Scientific keywords**	**Emotional keywords**
statistical	to calm
likelihood	to please
percentage	to worry
study	fortunately
to confirm	to encourage
scientific	doubt
single case study	anxiety
standardized	concerns
evident	insecure
significant	to feel
outcome	to unsettle
normal population	sorrow
to verify	courage
offering evidence	unscrupulous
statistic	fears
proven	insecurity
standard	feeling
verifiable	worried
to prove	
